# Methods for health surveys in difficult settings: charting progress, moving forward

**DOI:** 10.1186/1742-7622-4-13

**Published:** 2007-06-01

**Authors:** Kristof Bostoen, Oleg O Bilukha, Bridget Fenn, Oliver W Morgan, Clarence C Tam, Annemarie ter Veen, Francesco Checchi

**Affiliations:** 1 Department of Infectious and Tropical Diseases, London School of Hygiene and Tropical Medicine, London, UK; 2 International Emergency and Refugee Health Branch, Division of Emergency and Environmental Health Services, National Center for Environmental Health, Centers for Disease Control and Prevention, Atlanta, Georgia, USA; 3 Department of Public Health and Policy, London School of Hygiene and Tropical Medicine, London, UK; 4 East of England Regional Epidemiology Unit, Health Protection Agency, UK; 5 Department of Epidemiology and Population Health, London School of Hygiene & Tropical Medicine, London, UK

## Abstract

Health surveys are a very important component of the epidemiology toolbox, and play a critical role in gauging population health, especially in developing countries. Research on health survey methods, however, is sparse. In particular, current sampling methods are not well adapted for certain 'difficult' settings, such as emergencies, remote regions without easily available sampling frames, hidden and vulnerable population groups, urban slums and populations living under strong political pressure. This special issue of *Emerging Themes in Epidemiology *is entirely devoted to survey methods in such settings, and builds upon a successful conference in London highlighting problems with current approaches and possible ways forward. Greater investment in research on health survey methods is needed and will have beneficial effects for populations in need.

## 

Health surveys are the stethoscope, thermometer and pressure gauge of global health. Measurement of the health-based Millennium Development Goals depends on large-scale surveys such as the Demographic and Health Surveys, Multiple Indicator Cluster Surveys, and Living Standard Measurement Surveys [1]. For most international health interventions, including preventive disease control, curative care, health system strengthening, and emergency relief, population surveys are necessary to monitor implementation. Surveys can also provide direct measures of health outcomes and impact at the population level, and highlight important differentials in exposures and/or disease risk within particular groups, thus providing a trigger for action.

Despite the contribution that survey data can make to global health improvement, research to develop survey methods in difficult settings has largely stagnated over the past two decades. A mere handful of studies on this topic have been published. This may be because of a perception that surveys do not require the same sophistication and rigour as other types of studies, such as clinical trials. Yet surveys present a number of technical challenges, including the need to select representative samples, achieve adequate statistical precision and minimise bias in data collection. In resource-rich, industrialised settings, the surveyor's task is mostly straightforward: here, situations are often stable; communities are administratively organised; people are largely familiar with the use of surveys; transport and logistics are not problematic; capacity for data collection and analysis is high; legal and socio-economic conditions tend to protect participants against the untoward effects of research; and, crucially, comprehensive, stable population lists are more readily available, allowing researchers to select a representative random sample, the gold standard of survey sampling. Furthermore, the existence of sophisticated health information systems relying on prospective surveillance, and the high utilisation of health services, often remove much of the need for surveys, at least as a tool for monitoring service coverage and health outcomes.

There are, however, many settings throughout the world where these conditions are not met and where the problems of imprecision and bias are compounded by formidable logistical challenges, as well as serious political, security, cultural or ethical constraints. A list of such "difficult" settings might include: humanitarian crises resulting from conflict and natural disasters; poor and/or remote developing country settings where survey design options are constrained by insufficient census or geographic data; "hidden" and/or vulnerable populations (such as sex workers, orphans, street children, victims of sexual and gender-based violence, undocumented migrants, nomadic communities, and women as a whole in some cultures); urban and peri-urban slums and other marginalised areas in developing country cities; and populations under strong political pressure, among whom data collection may be actively discouraged by authorities and/or entail considerable risks for beneficiaries and researchers. Paradoxically, it is precisely in these settings that surveillance data are most lacking, and surveys most badly needed to generate information about population health.

On 15 February 2006, the London School of Hygiene and Tropical Medicine (LSHTM) hosted its first international conference on health survey methodology in difficult settings [2]. The conference was attended by 125 participants from 31 institutions, including academic centres from Europe, United States of America and Australia, international non-governmental organisations (NGOs), United Nations agencies, and major public health institutions.

This special issue of *Emerging Themes in Epidemiology *is an outcome of the conference and has been developed with support from LSHTM and the Centre for Research on the Epidemiology of Disasters (CRED), Université Catholique de Louvain, Belgium. It represents a move to rekindle international interest in methodological aspects of health surveys. The issue showcases recent survey-related work in a variety of health-related fields, and encourages inter-disciplinary sharing of experience in an Open Access internet publication format.

Several contributions to this issue come from the humanitarian relief community. Over the past 30 years, surveys have been increasingly used for assessing, monitoring and guiding emergency operations in settings affected by conflict and natural disasters. In these settings, uncertain and rapidly-changing sampling frames are common, working conditions are challenging, data collection is not considered a priority, and political sensitivities abound. In his opinion piece, Spiegel [3] considers the role of various humanitarian stakeholders (NGOs, United Nations agencies, and academic centres) in the implementation of surveys in such conditions, and offers recommendations for how to improve existing practices through standardisation of methodologies, better training for field staff, timely deployment of skilled epidemiologists, and inter-agency peer review. Degomme and Guha-Sapir [4], from CRED, reflect on the creation of a database of surveys conducted in emergencies, and explore it to describe and interpret recent global trends.

Prudhon and Spiegel [5] review the validity of more than 350 mortality, nutrition and vaccination coverage surveys conducted during the last decade. This review both updates and improves upon previous work on nutritional [6] and HIV serological and behavioural surveys (Paul Spiegel, unpublished data), offering a much-needed reality check on the quality of survey work. No health topic is as fundamental as mortality and, in crises, its measurement is of crucial importance for both operational planning and advocacy. Moreover, as shown by recent work in Darfur [7] and Iraq [8], such surveys can be politically as well as methodologically controversial. Although manuals and guidance exist, survey methods used to estimate mortality retrospectively are only partially validated, and a number of methodological questions remain outstanding. The Working Group for Mortality Estimation in Emergencies [9] highlights several of these and suggests a set of best practice procedures.

These first four papers could not be more timely given the current drive to establish a global system to track the evolution of major crises through the systematic implementation of mortality and nutrition surveys [10]. The bottom line is that, while the quality of humanitarian surveys is improving, progress is slow and demand for data considerably outstrips present capacity. As a start, where guidelines exist, they should be adhered to more rigorously and adequate resources must be set aside to allow for sound data collection.

There are nonetheless many situations for which existing data collection methods do not offer feasible solutions and these often concern the most vulnerable and deprived populations. Approaches to deal with the lack of adequate sampling frames are painfully limited and have advanced little in the past decades. Traditionally, the main solution has been cluster sampling, whereby a representative number of starting points is selected within the target population based on probability proportional to size. Individuals or households around these points are then included using a variety of sampling methods. The standard 30 × 7 and 30 × 30 cluster designs, with household selection performed according to the Expanded Programme on Immunisation (EPI) method (perhaps more familiar to readers as "spin-the-pen" [11]), has been adopted widely, usually without sufficient appreciation of its limitations. This formulaic approach often leads to neglect of appropriate sample size calculation (i.e. considering the optimal number of clusters and households) and insufficient recognition of the need to plan for the effect of clustering (i.e. the design effect) and account for this in the analysis.

Despite its popularity, the EPI method is fraught with potential selection biases (such as favouring denser areas and households around the starting point) [12] and can be particularly difficult to conduct in urban and peri-urban settings. This is clearly a major area where alternative approaches need to be developed urgently. Grais et al.'s report from Niger [13] offers promising improvements to the "spin-the-pen" selection of households in urban areas. Bostoen et al. [14] take a more fundamental approach, and explore the use of mathematical programming as a tool for optimising household sampling designs. They use the example of population estimation, a key prerequisite for meaningful health planning in any setting without reliable census data. Making these alternative techniques user-friendly, and widely disseminating the skills for their application in the field should be a priority.

The concluding papers in our issue exemplify forward-thinking approaches to survey design and implementation, of the kind that we hope will increasingly inform health research in developing countries. Vallée et al. [15], working in Lao People's Democratic Republic, question the inevitability of cluster sampling based on probability proportional to population size, especially when the goal is to explore geographic determinants of health. Instead, they propose a purposeful selection of clusters guided by knowledge of the spatial arrangement of key population characteristics. Shirima et al. [16] describe their experience with personal digital assistants in a large, multi-indicator baseline survey in rural Tanzania and show that the use of advanced technologies can greatly simplify and facilitate the work of survey teams. Unfortunately, these devices still remain beyond the reach of many organisations, and require advanced expertise not often available on the ground. Nonetheless, they are a promising tool for data management in the future. Finally, Hargreaves et al. [17] report on a study from rural South Africa that compared standard survey approaches to a participatory ranking exercise as methods for rapidly estimating household wealth, a key determinant of health status. Although their results are not definitive, this study is a fitting conclusion to our issue, as it suggests that traditional survey methods need not always be put forward as a default solution. Innovative tools that partly incorporate participative and qualitative elements may be more appropriate in some settings.

Taken together, this collection of papers is a small but important leap towards greater investment in health survey methodology in settings where it is most needed. Following the success of the first conference in 2006, CRED and LSHTM will be co-hosting a second conference, to be held in Brussels on 4–5 June 2007 [18]. Much remains to be done, however. There is, in particular, much scope for the development of innovative approaches through collaboration with other disciplines, such as ecology, that have expertise in survey methodologies. Such inter-disciplinary collaboration should aim to convert potential methods into practical field tools, including reference and training materials for implementing agencies. Moving this agenda forward will undoubtedly require greater funding for both academic and operational research. Advocacy is needed to champion these activities among donors, governments and public health practitioners. While better survey data are crucial for governments, relief agencies and donors, they must ultimately serve to benefit the affected populations. Decision-making based on imprecise and biased data generated by insufficiently funded and skilled data collectors risks jeopardising health improvements. If there is an international obligation to equitably provide health to human beings, and if robust data are indispensable for health planning, then it is clear that provision of health services to many populations is being hindered by the use of sub-optimal survey techniques. Greater investment in the development of survey methods, both financially and intellectually, is urgently needed if major organisations are to target, monitor and evaluate their programmes more effectively.

## Competing interests

The authors declare that they have no competing interests.

## Authors' contributions

All authors were part of the editorial committee for the special issue of *ETE *on health survey methods in difficult settings, and contributed to the writing of this editorial. K Bostoen and F Checchi co-wrote the first draft of this editorial, and, along with C Tam, coordinated work on the special issue. All authors read and approved the final manuscript.
